# Supplementary material for: MetaRibo-Seq measures translation in microbiomes
Source: Nat Commun. 2020 Jun 29;11:3268. doi: 10.1038/s41467-020-17081-z (PMC7324362; doi:10.1038/s41467-020-17081-z)
Supplement: Supplementary file 10 — Supplementary Data 7 [file 41467_2020_17081_MOESM10_ESM.zip › File2/Confidence_High_Taxonomy/260453_out.krona.html]

Javascript must be enabled to view this page.

members
magnitude
magnitudeUnassigned
count
unassigned
taxon
rank

260453\_out

25

superkingdom
25
2

25
phylum
1239

186801
25
class

25
order
186802

25
family
541000

1263
genus
25


SRS012273\_contig\_number\_2901SRS013098\_contig\_number\_44384SRS013951\_contig\_number\_5757SRS014235\_contig\_number\_28453SRS015663\_contig\_number\_33887SRS015890\_contig\_number\_13352SRS017916\_contig\_number\_4421SRS018984\_contig\_number\_19056SRS019685\_contig\_number\_35526SRS020233\_contig\_number\_2899SRS024492\_contig\_number\_12144SRS050752\_contig\_number\_4037SRS051031\_contig\_number\_24835SRS052697\_contig\_number\_32332SRS062701\_contig\_number\_4941SRS063985\_contig\_number\_12249SRS074964\_contig\_number\_contig-100\_11.35311SRS075821\_contig\_number\_1044SRS098571\_contig\_number\_13417SRS104197\_contig\_number\_contig-100\_259.3309SRS142542\_contig\_number\_26036SRS143181\_contig\_number\_13556SRS146764\_contig\_number\_6672SRS148159\_contig\_number\_23655SRS971275\_contig\_number\_36403
46228
25
species
